# Repairing quite swimmingly: advances in regenerative medicine using zebrafish

**DOI:** 10.1242/dmm.016352

**Published:** 2014-07

**Authors:** Wolfram Goessling, Trista E. North

**Affiliations:** 1 Brigham and Women’s Hospital/Dana-Farber Cancer Institute, Boston, MA 02215, USA.; 2 Harvard Medical School, Boston, MA 02115, USA.; 3 Harvard Stem Cell Institute, Cambridge, MA 02138, USA.; 4 Beth Israel Deaconess Medical Center, MA 02115, USA.

**Keywords:** Regeneration, Zebrafish, Disease model, Gastrointestinal, Hematovascular

## Abstract

Regenerative medicine has the promise to alleviate morbidity and mortality caused by organ dysfunction, longstanding injury and trauma. Although regenerative approaches for a few diseases have been highly successful, some organs either do not regenerate well or have no current treatment approach to harness their intrinsic regenerative potential. In this Review, we describe the modeling of human disease and tissue repair in zebrafish, through the discovery of disease-causing genes using classical forward-genetic screens and by modulating clinically relevant phenotypes through chemical genetic screening approaches. Furthermore, we present an overview of those organ systems that regenerate well in zebrafish in contrast to mammalian tissue, as well as those organs in which the regenerative potential is conserved from fish to mammals, enabling drug discovery in preclinical disease-relevant models. We provide two examples from our own work in which the clinical translation of zebrafish findings is either imminent or has already proven successful. The promising results in multiple organs suggest that further insight into regenerative mechanisms and novel clinically relevant therapeutic approaches will emerge from zebrafish research in the future.

## Introduction

Regenerative medicine offers the promise of regaining organ function after acute or chronic injury. Regenerative approaches aim to promote, enhance and re-establish organ-specific repair processes to reconstitute organ structure and function after injury or in the setting of disease progression or treatment. Currently, the impact of the field of regenerative medicine in clinical practice is limited to a few specialized although highly successful practices, such as autologous bone marrow transplantation (Clift et al., 2004), partial liver transplantation (Vagefi et al., 2011) and skin grafting (Markeson et al., 2013). In many other scenarios, however, replacement of the damaged tissue or organ function has been the more commonly used approach, be it through the use of a specialized peptide (such as insulin), advanced machines (kidney dialysis) and manufactured support (pacemakers, joint replacements or prosthetics), or solid organ transplantation (heart, lung, liver, allogeneic bone marrow transplantation). Intensive research is focused on the discovery and isolation of specialized cell types (e.g. embryonic or adult stem cells), or small molecules that can boost the innate ability of the human body to achieve endogenous regenerative repair for a variety of tissues; significant effort is likewise being put forth to develop methods to stimulate repair in organ systems in which no intrinsic regenerative process is currently evident in humans.

Over the past three decades, studies using zebrafish have been very successful in enhancing our understanding of the principles of early vertebrate development and organogenesis. These investigations have revealed the iterative use of key signaling pathways involved in germ layer development, axis formation, and the specification and differentiation of mature organs. The first use of fish as a scientific model organism, however, was in the field of regenerative medicine: Broussonet demonstrated the regeneration of the pectoral fins of goldfish in 1786, and Thomas Hunt Morgan studied regeneration of amputated fish fins in 1901, as described in his book *Regeneration* (Sunderland, 2010), just prior to his seminal studies in fruit flies that established the field of modern genetics.

The regenerative potential of most mammalian organs and tissues can be classified broadly into those that regenerate well and almost constantly, such as blood, intestine and skin; those that can regenerate well after injury, such as liver, skeletal muscle and bone; and those that are commonly believed to have low regenerative potential: heart, kidney, pancreas and neural tissue. Although the molecular and cellular conditions enabling or limiting regeneration of these organs are not known, it is well described that scar formation with the deposition of fibrotic tissue is one factor, probably among others yet to be identified, that can severely impair the regenerative potential of any tissue. In contrast to many invertebrate models, which are well known for their ability to regrow a variety of injured body parts [e.g. planaria (Elliott and Sánchez Alvarado, 2013)], it was long thought that higher vertebrate species, such as humans, had more limited capabilities for organ repair. However, it is now widely appreciated that many vertebrate model organisms, such as axolotls (McCusker and Gardiner, 2011) and tadpoles (Slack et al., 2008), also have exquisite regenerative capabilities, albeit only for specific organ systems. In recent years, zebrafish in particular have been used to elucidate mechanisms of organ repair both in tissues that we now appreciate possess strong regenerative capacity in mammals, such as blood and liver, and in tissues that do not, including the fins (limbs), heart and brain, which we will describe in more detail below (Sánchez Alvarado, 2006; Muneoka and Bryant, 1982; Mochii et al., 2007). These findings have enhanced our understanding of the cellular and molecular mechanisms involved in organ repair, showing striking conservation of genetic regulation across organ systems (Congdon et al., 2008; Lien et al., 2014; Yang et al., 2014), as well as between vertebrate and invertebrate species (Lin et al., 2008; Petersen and Reddien, 2009; Philipp et al., 2009; Srivastava et al., 2014), as exemplified by the WNT signaling pathway. More recently, chemical genetic screens applied in conjunction with clinically relevant injury models have led to translational efforts aimed at the introduction of an array of novel therapeutic options for the field of regenerative medicine for problems as diverse as hearing loss, kidney or liver injury, and bone marrow transplantation (Esterberg et al., 2013; North et al., 2010; North et al., 2007; Sanker et al., 2013). This Review highlights the utility of the zebrafish model in aiding progress toward these goals and the subsequent application of novel therapeutic approaches to the field of regenerative medicine, based on our own experiences using the hematovascular and gastrointestinal systems as illustrative examples (Cox et al., 2014; Cutler et al., 2013; Goessling et al., 2011; North et al., 2007).

## The therapeutic potential of zebrafish research for regenerative medicine

The therapeutic potential of zebrafish as a model for organ development and disease has been demonstrated in many organ systems. Large chemical mutagenesis screens using the potent mutagen *N*-ethyl-*N*-nitrosourea (ENU) identified essential genes, and gave insight into the physiology and pathophysiology of a variety of disease states. These mutants also frequently highlighted the genetic diversity contributing to morphologically similar disorders, potentially providing insight into the varied responses to current therapeutics or providing a platform for rational development of treatment options. For example, many diseases affecting blood formation and red blood cell physiology have been either explained by or modeled in zebrafish mutants, including the identification of previously uncharacterized genetic defects causing human disease (Donovan et al., 2000; Donovan et al., 2005; Sham et al., 2005; Chen et al., 2010; Wang et al., 2011b). Similar outcomes have occurred in a diverse array of other organ systems; other forward-genetic screening methods, such as mutagenesis via viral insertions (Amsterdam et al., 1999) or use of transposon-mediated gene disruption (Kotani et al., 2006), have yielded equally important and clinically applicable findings, which are reviewed in detail elsewhere (Jing and Zon, 2011; Patton and Zon, 2001). These studies are relevant to the field of regenerative medicine because they reveal insights into the genes and signaling cascades and cellular networks that are important for creating both the structure and function of each organ system. Therefore, developmental studies, including the characterization of zebrafish mutants, can provide a roadmap or blueprint to re-establish or augment cellular differentiation or tissue function to aid regenerative repair *in vivo*, or *in vitro* for the stepwise production and expansion of stem and progenitor populations for therapeutic cellular replacement strategies. One example of this in practice is the use of the soluble factor Activin A to induce definitive endoderm *in vitro* from pluripotent stem cells, based in part on the established role of nodal signaling in endoderm specification during embryogenesis discovered in zebrafish (Schier, 2003).

More recently, induction of targeted mutations in the zebrafish genome has enabled focused studies aimed at validating disease relevance and/or mechanism of effect for select genes of interest, particularly with regard to those already associated (but not necessarily identified as causal) with disease phenotypes, including many tumor suppressors and oncogenes. The first iteration of these ‘reverse genetic’ approaches, known as TILLING (targeting induced local lesions in genomes) (Wienholds et al., 2003), took advantage of the potent mutagenic activity of ENU (used in the forward-genetic screens) and the headway made in sequencing the zebrafish genome (Howe et al., 2013). Many of these studies have produced zebrafish embryos with remarkable phenotypic correlations with the associated clinical manifestations of the human disease mutation. For example, zebrafish heterozygous for a mutation in adenomatous polyposis coli (APC), a central regulator of β-catenin stability and Wnt signaling, develop intestinal tumors comparable to the intestinal polyposis in humans with a corresponding *APC* mutation at the genotypic and phenotypic level (Haramis et al., 2006). Further studies revealed roles for APC in liver development (Goessling et al., 2008), as well as in the propensity for endodermal tumor formation (Haramis et al., 2006). These insights can be used to model hepatoblastoma development, and to design rational treatments to block cancer progression and recover hepatic function (Goessling et al., 2007). Although these are not specific models of organ injury in need of regenerative repair, the mutants produced by either random or induced mutations provide valuable tools to investigate the repair or recovery of specific aspects of tissue function, including the identification of compound modifiers applicable to regenerative medicine.

The fact that the development of zebrafish embryos occurs *ex utero* enables efficient introduction of foreign nucleic acids by microinjection. Long used as an effective means to ‘rescue’ mutant phenotypes to prove causation and produce transgenic fluorescent-tagged reporters of select genes of interest, microinjection has also enabled both transient and targeted gene knockdown. Although not without the caveats of potential nucleotide toxicity or off-target effects, morpholino oligonucleotide (MO) injection has been effectively used to antagonize translation in an antisense manner, thus blocking or diminishing protein production and revealing gene function. Furthermore, the procedure is titratable, providing the ability to bypass phenotypes associated with early embryonic lethality. For example, in zebrafish, just as in mice, complete loss of Wnt signaling (Haegel et al., 1995; Huelsken et al., 2000; Morkel et al., 2003; Weidinger et al., 2005) is lethal at early stages of embryonic development. However, partial antagonization of gene expression has identified a role for Wnt signaling in other organ systems with later developmental requirements, such as the liver (Goessling et al., 2008). Transient knockdown can be combined with ENU or TILLING mutations to eliminate functional redundancy or perform epistasis experiments. More recently, a series of studies have taken advantage of the speed of transient knockdown to assess the functional relevancy of candidate disease genes identified through patient genome-wide association studies on a variety of clinical phenotypes, including platelet production (Gieger et al., 2011), chronic kidney disease (Liu et al., 2011; Pattaro et al., 2012) and liver function (Liu et al., 2013). All these investigations revealed several genes that affect organ development, function and/or susceptibility to disease; this information can be used to conduct further chemical and/or genetic interaction or suppressor screens in zebrafish or to guide follow-up studies in mammalian models. As many of these genes correlate with loss of specific aspects of organ production or function in humans, knowledge of these novel regulatory genes and/or small molecular modifiers of their activity could considerably impact the development of targeted therapeutics relevant to regenerative repair. The advent of next-generation genome editing methods, such as TALENs (Bedell et al., 2012) and CRISPR/Cas9 (Hwang et al., 2013), in combination with the zebrafish mutation project (Kettleborough et al., 2013), will enable the widespread introduction and continued use of disease-relevant zebrafish mutants for clinically directed research, including studies in the field of regenerative medicine.

## Chemical genetic screening for regenerative therapeutics

Chemical screens are now widely used to interrogate biological processes from the earliest stages of development to organogenesis and organ function. They have provided insight into the pathophysiology of diseases and led to the discovery and clinical application of novel treatment strategies. As such, they represent the most *direct* opportunity to translate findings from the ‘fish-tank to the bedside’. In general, chemical screens can serve two different purposes: they can reveal novel chemical structures that target a particular aspect of a cellular process or bind to defined regulatory molecules, or they can inform about the genetic mechanisms controlling a given biological process. The former typically involves tens of thousands of novel compounds, often of unknown mechanism, and is frequently performed by or in collaboration with the pharmaceutical industry. The latter, which has become increasingly popular in the zebrafish field, is to use a limited number of well-annotated chemicals, so-called ‘known bioactives’, whereby some aspect of the mechanism of action of a particular compound is characterized, allowing one to identify the molecular pathways that might be involved in the modulation of a specific phenotype of interest. If the developmental phenotypes used for screening are functionally related to organ growth or differentiation, the compounds isolated might have a direct impact on relevant aspects of regenerative capacity.

Chemical screens in the zebrafish were pioneered initially to identify compounds that could alleviate a mutant phenotype, which was often relevant to a human disease state (Zhong et al., 2000; Peterson et al., 2004; Stern et al., 2005). Subsequent studies were designed to identify novel compound regulators of well-conserved aspects organ formation, typically using chemical libraries composed of aforementioned bioactives (Garnaas et al., 2012; Andersson et al., 2012). One of the first screens to successfully use this approach was aimed at the identification of modulators of hematopoietic stem cell (HSC) formation, as detected by *in situ* hybridization for expression of the well-characterized HSC markers *runx1* and *cmyb* (North et al., 2007). After screening ~2500 compounds, this study revealed a number of relevant HSC regulators, including prostaglandin E2 (PGE2) (discussed in more detail below) and nitric oxide (NO) (North et al., 2009), both of which showed strong conservation of effect *in vitro* and *in vivo* across vertebrate species, in developmental regulation and organ regeneration. The fact that many libraries of known bioactives contain FDA-approved or biologically characterized drugs might not only aid the identification of the mechanism of action for further investigation, but also facilitate translation into clinical practice.

More recent chemical screens have moved away from gene-associated modulation to look directly at impact on abnormal organ function or tissue physiology. For example, Poss and colleagues performed a chemical screen for compounds that alter the proliferation of cardiac myocytes during heart development (Choi et al., 2013). They identified several small molecules, acting through the hedgehog, insulin-like growth factor and transforming growth factor β signaling pathways, that modulate heart regeneration after mechanical or genetic ablation injuries. Similarly, in a zebrafish model of acetaminophen-mediated liver toxicity that mimics the clinical picture of acute human liver failure, North et al. used a chemical screening approach to identify PGE2 as a pro-proliferative compound that facilitates liver regeneration and reacquisition of hepatic function after injury, enhancing survival in the embryo and adult (North et al., 2010). Together, these studies highlight the potential translational impact of chemical screening to identify regulators of organ formation and physiology, with application to therapeutic regeneration of tissue structure or function after injury or in disease states.

## Regenerative assays in zebrafish

There are two primary objectives for conducting regenerative assays in zebrafish, which differ depending on whether the regenerative response is conserved in mammals or not. The first approach is to study organs that undergo regenerative repair in zebrafish, but do not regenerate well, if at all, in adult mammals. Here, the primary goal is to elucidate the principal cellular and molecular mechanisms regulating the process, and then compare these with pathways initiated in the response to injury in mammals. This would give some understanding of the primary components that can be used to drive each phase of regeneration of the organ or tissue of interest. This insight could then be used to develop approaches to improve regeneration in mammalian models, and eventually in patients, by chemical or genetic means. These studies greatly benefit from the classic genetic strengths of the zebrafish, including the ease of genetic manipulation, targeted gene expression or ablation, and innovative methods to induce relevant injuries.

The second approach is to utilize zebrafish to study organs that are known to regenerate well in mammals, such as the bone marrow or liver. In this case, therapeutically relevant assays have been established in mammals, and many of the signals controlling the regenerative process and some methods for intervention have been defined. If a technically feasible and clinically consistent injury model is available, then the goal would be to use zebrafish to identify new therapeutic opportunities by combining the known conservation of the regenerative process in teleosts with *in vivo* screening for chemical or genetic modulators of the primary response. Several physiologically relevant assays have now been established in zebrafish that can be used either to screen modifiers directly or for translational testing of novel compounds identified in developmental regulatory screens. As more studies attempt to replicate standard regenerative assays from mammalian models in the zebrafish system, we find remarkable conservation not only of genetic regulation but also of clinical parameters, known biomarkers and, perhaps most surprisingly, time to recovery. This suggests that novel modifiers discovered through zebrafish regenerative assays might have translational benefits.

## Studies exploiting differences in regenerative potential between fish and humans

### Fin regeneration

As noted above, the first regenerative studies in fish date back to the 18th century, focused on the fin in goldfish. Fin studies were also the first to assess the regenerative usefulness of the zebrafish model: in 1995, Johnson and Weston performed a genetic screen for temperature-sensitive mutations that affect tailfin regeneration in adult zebrafish (Johnson and Weston, 1995), which subsequently led to the identification of several key genetic regulators of this process (Makino et al., 2005; Nechiporuk et al., 2003; Poss et al., 2002a; Whitehead et al., 2005). The mutant lines serve as models for understanding vertebrate limb regeneration, as the fin is repaired after amputation by formation of a blastema that contains progenitor cells, which facilitate the coordinated growth and differentiation of multiple cell types. It is hoped that the insights gained from fin regeneration studies could lead to novel therapeutic approaches to stimulate aspects of limb repair in humans. Limb regeneration, including that of the fin, occurs via a stepwise mechanism after injury (Stoick-Cooper et al., 2007a). Although the spatio-temporal dynamics of the process can vary significantly across species, the central genetic regulators of the regenerative process are well conserved. Furthermore, through targeted approaches, essential signaling cascades, such as the retinoic acid (White et al., 1994) and Wnt (Stoick-Cooper et al., 2007b) signaling pathways, identified in the fin studies as primary regulators of the regenerative response, are likewise now appreciated to be essential for regeneration in other organ systems, indicating the presence of core signals that stimulate and/or drive crucial aspects of tissue repair.

### Cardiac regeneration

Cardiac regeneration studies in the zebrafish, pioneered by Poss and colleagues, demonstrated for the first time that a vertebrate heart was indeed capable of regeneration; surgical resection of ~20% of the apex of the cardiac ventricle can be repaired over the course of two months (Poss et al., 2002b). This is in contrast to the hearts of an adult mammal (mouse, human), for which no significant regeneration had been observed following cardiac injury, although recent reports suggest that some facets of cardiac repair in mice might be derived from pre-existing cardiomyocytes (Senyo et al., 2013). In recent years, a number of other injury models have joined the surgical resection model, each of which has specific advantages. The induction of cryoinjury causes necrosis of cardiomyocytes, which stimulates subsequent repair over 2–4 months, thereby more closely resembling certain aspects of myocardial infarction (Chablais et al., 2011; González-Rosa et al., 2011; Schnabel et al., 2011). In addition, genetic ablation achieved by expression of diphtheria toxin A in heart muscle cells can result in ~60% cell necrosis and symptoms of advanced heart failure (Wang et al., 2011a). Finally, Chi and colleagues targeted cardiomyocytes in the ventricle of zebrafish embryos through a genetic ablation method using the bacterial enzyme nitroreductase; upon exposure to the antibiotic metronidazole, nitroreductase converts the drug to a cytotoxic DNA-crosslinking compound, causing extensive cell death. This assay revealed that embryonic cardiomyocytes in the atrium can reacquire a progenitor-like state to achieve cardiac regeneration in a Notch-dependent fashion (Zhang et al., 2013). Together, these studies demonstrate the breadth of resources currently available in the zebrafish model, as well as the speed of continuing evolution in the field of heart regeneration, a process that until recently was essentially impossible to assay in traditional mammalian models (Senyo et al., 2013). The zebrafish cardiac injury models provide a means to guide the search for cell types and pathways that can be driven to aid regenerative repair of cardiac function in mammals, and identify chemical modifiers to limit damage and/or stimulate regeneration across species. Ultimately, these investigations could contribute to the discovery of novel drug targets or candidate compounds that could enhance cardiac repair in patients with heart failure or after acute myocardial infarction.

### Brain regeneration

Recent studies using novel approaches to surgically induce lesions in the teleost telencephalon have provided new insights into the regenerative potential of neural tissues. A variety of approaches have been performed to induce injury, as extensively reviewed recently (Kizil et al., 2012). The most common procedure utilized to study brain regeneration is the production of physical lesions by disruptive force, which leads to a complex response involving multiple cell types. Using a physical injury model, radial glia lining the cerebral ventricles were shown to proliferate and produce progenitor cells that form differentiated neurons (März et al., 2011). However, despite that promising outcome, secondary effects of the traumatic injury, including inflammation and disturbance of the blood-brain barrier, have the potential to impede both the interpretation of outcomes and the identification of the cellular and genetic modulators of the regenerative process. Other more targeted forms of injury include chemical-mediated noxic stimuli, such a triethyltin, somatostatin and methylmercury (reviewed in Kizil et al., 2012). More recently, as in the heart, genetic ablation methods have likewise been employed to induce targeted cell injury, such as the use of the nitroreductase system in the retina (Fraser et al., 2013). These studies illuminate the potential of zebrafish models to decipher basic principles of neural regeneration after injury, which cannot be readily studied in mammalian systems.

## Studies exploiting the similarities in regenerative potential between fish and humans

### Hematopoietic regeneration

As in mammals (Thomas, 1964), regeneration assays in the zebrafish hematopoietic system were initially performed in irradiation-induced injury models (Traver et al., 2003). Traver demonstrated that the blood cell populations in the kidney marrow, the site of adult hematopoiesis in the zebrafish, can be characterized by straightforward size [forward scatter (FSC)] and granularity [side scatter (SSC)] profiling through flow cytometry (Traver et al., 2003); this method was later applied to evaluate irradiation-mediated marrow damage and establish ablation thresholds (Traver et al., 2004). In subsequent studies, exposure to sublethal doses of gamma irradiation enabled assessment of genetic modulation of autologous hematopoietic recovery after injury (Burns et al., 2005). Importantly, hematopoietic homeostasis was reestablished by stem and progenitor proliferation and differentiation over the course of 2 weeks, consistent with mammalian models. This same protocol was also utilized to evaluate conservation of HSC function for chemical screen hits between embryonic and adult zebrafish (Goessling et al., 2009; North et al., 2007). Further elevation of the radiation dose allowed hematopoietic regeneration by adult-to-adult HSC transplantation (Traver et al., 2003; Traver et al., 2004); this technique, an important therapeutic approach for leukemia and lymphoma, is used to determine the presence of a true long-lived multipotent HSC in mammalian models. Use of transgenic lines and application of standard dye-efflux assays (side population) confirmed that long-lived HSCs and lineage progenitors were kidney marrow derived (Kobayashi et al., 2008; Langenau et al., 2004). It is currently estimated by limiting dilution transplantation analysis that the zebrafish kidney marrow contains ten functional HSCs (Hess et al., 2013). Transplantation methodologies were further improved by the generation of *casper* fish, which lack pigmentation, enabling *in vivo* visualization of HSC homing, engraftment and chimerism (White et al., 2008) and the development of MHC-matched lines to prevent donor cell rejection (de Jong et al., 2011). Subsequent studies have also tested the utility of 5-fluorouracil (5-FU)-mediated myeloablation of the kidney marrow and examined the factors influencing hematopoietic homing following transplantation, determining that each response is conserved with mammals (Glass et al., 2013; Tsinkalovsky et al., 2007). Together, these assays, which closely align with both murine and human regenerative protocols, provide useful ways to test pharmacological modifiers of donor or host responses during the regenerative process. They can also be used to evaluate the functional kinetics and interactions of stem and progenitor populations as they repopulate the hematopoietic system in real time. Therefore, conducting these assays in the zebrafish model might be useful for expanding our current methods for the treatment of human hematologic disorders or diseases.

### Liver regeneration

Just like the blood, the liver regenerates robustly after injury and mammalian models for partial hepatectomy have existed for more than 80 years (Higgins and Anderson, 1931). A similar surgical resection technique can be effectively applied to adult zebrafish: the zebrafish liver consists of three lobes, one ventral and two lateral, and local regrowth is observed after resection of a single lobe. Although the amount of liver removed (approximately one third) in zebrafish assays is in contrast to rodent studies, in which typically two-thirds of the liver is resected, the cellular mechanism of repair, hepatocyte proliferation, appears to be highly conserved (see Table 1). Attempts at two-thirds partial hepatectomy in zebrafish led to overwhelming mortality, with no animals surviving for two days (Kan et al., 2009) after resection; however, it remains unclear if that discrepancy represents a functional difference or simply a technical hurdle. Sadler et al. found that the cell cycle regulator *uhrf1*, mutated by viral insertion, was an important regulator of both embryonic liver growth and regeneration after surgically mediated partial hepatectomy: adult *uhrf1^+/−^* fish have reduced regrowth of the resected lobe (Sadler et al., 2007). Goessling et al. describe a similar technique using ultrasound volumetric analysis and length measurements to quantify regrowth, and reveal parallel regenerative kinetics between the liver of zebrafish and mice, taking 5–7 days for full recovery after surgical resection (Goessling et al., 2008). Using *APC* mutant zebrafish and mice, that study likewise illustrated the conserved functional importance of Wnt signaling (Decaens et al., 2008) during embryonic and adult liver growth across species, with follow-up investigations demonstrating maintenance of regulatory interactions between PGE2 and the Wnt signaling pathways in vertebrate regeneration (Goessling et al., 2009). Subsequent resection studies performed by several groups have shown a role for *topoisomerase 2a* (Dovey et al., 2009) and for both fibroblast growth factor (FGF) and bone morphogenic protein (BMP) signaling in optimal liver repair (Kan et al., 2009). Together this robust surgical model serves as a foundation for screening for novel chemical and genetic modifiers of liver regeneration, particularly applicable to resection due to cirrhosis or cancer in the clinical setting.

**Table 1.**
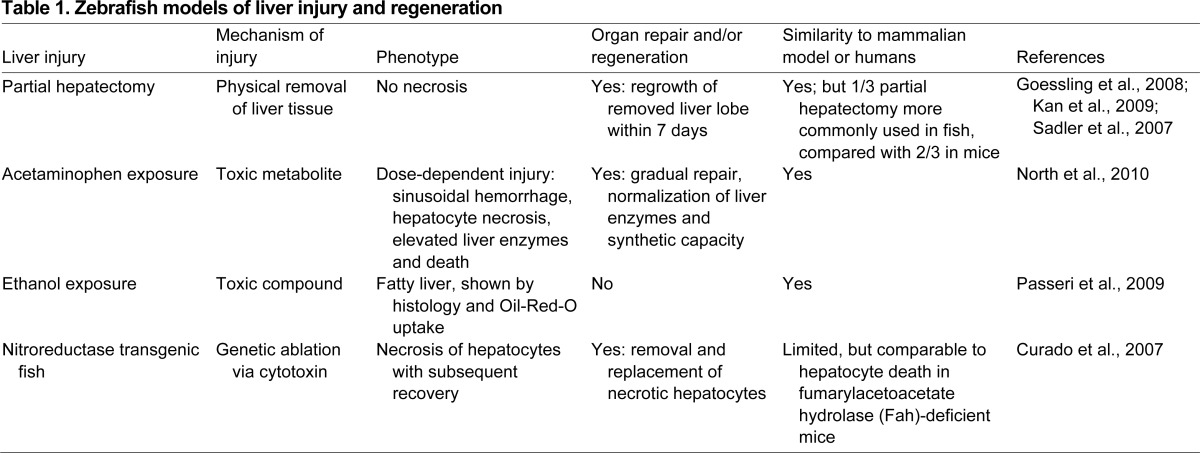
Zebrafish models of liver injury and regeneration

In contrast, other zebrafish models of liver regeneration have focused on the induction of hepatocyte injury and necrosis, rather than removal of tissue (see also Table 1). The first documented observation of hepatic regeneration in the zebrafish was toxicology mediated: following exposure to 4-chloroaniline, hepatic cytoarchitecture and ultrastructure, as well as survival, were affected in a dose-dependent manner (Burkhardt-Holm et al., 1999). Stainier and colleagues introduced nitroreductase-based genetic ablation in the liver (Curado et al., 2007; Curado et al., 2008); this approach has recently been used to elucidate the biliary origin of regenerating hepatocytes following almost complete loss of hepatocyte mass (Choi et al., 2014; He et al., 2014). These elegant studies reveal mechanisms of cellular recovery that could not be easily investigated in murine experiments, including fate mapping and direct visualization of the cellular contributions and movements occurring in the regenerative process. Sadler and colleagues have pioneered the use of ethanol exposure in zebrafish larvae to elicit the most common form of human liver damage; consistent with mammals, hepatic fat accumulation occurs as a direct and dose-dependent consequence of alcohol intake (Howarth et al., 2011). Finally, our group took advantage of the natural role of the liver in detoxification and developed a model for toxicity mediated by acetaminophen (APAP, also known as Tylenol^®^ or paracetamol) (North et al., 2010). APAP overdose is the most common cause of acute liver failure, and the response in zebrafish is highly similar to both murine models and human disease: APAP-treated zebrafish exhibit elevated liver enzymes, liver sinusoidal hemorrhage, hepatocyte necrosis, proteomic changes and dose-dependent death due to loss of liver function. This model was utilized for chemical screening, as described above, and led to the identification of PGE2 as a hepatoprotective compound that limits liver damage and increases survival (North et al., 2010). In this example, the conserved physiological response to APAP across species was a great advantage to then exploit the unique opportunity to perform an unbiased chemical screen in zebrafish that revealed a compound that could be directly used in mammalian models. PGE2 could be used in synergy with current treatment options of APAP liver toxicity to extend the window of therapeutic effectiveness and enhance repair, which might enable patients to avoid the need for liver transplantation in the clinical setting. In sum, these studies demonstrate the diversity of approaches that can be used to induce hepatic injury to study and/or modify the dynamics of liver regeneration.

### Other organ systems with regenerative potential in zebrafish

In addition to those discussed above, an increasing number of groups have utilized zebrafish to study regenerative repair after injury induced by a variety of methods in a broad array of organs, ranging from muscle repair after crush- or laser-induced injuries (Otten et al., 2012; Rodrigues et al., 2012; Seger et al., 2011) and retina regeneration after focused light injury (Ramachandran et al., 2011) to the pancreas following genetic ablation (Andersson et al., 2012; Moss et al., 2009; Pisharath et al., 2007) and scales (skin) after physical removal (de Vrieze et al., 2014). Together, these studies highlight the growing field of regenerative medicine in zebrafish, which will enable the elucidation of signals that make repair possible in those organs that typically do not regenerate in mammals, and identify novel molecules to directly enhance regenerative processes already utilized in clinical medicine today.

## Examples of clinical translation of zebrafish regenerative approaches

Although many zebrafish studies have the goal of therapeutic relevance, actual translational application of findings from zebrafish investigations is still in its infancy relative to mammalian models. That said, recent investigations stemming from chemical screening approaches focused on highly conserved aspects of regenerative biology of a select organ system, as discussed above, have directly shown the full potential of the zebrafish model for discoveries in the field of regenerative medicine. Here, we summarize the major attributes of two such studies from our own work. These investigations exploited conserved regenerative models (blood and liver) and a chemical screening approach, combined with the vast array of established tools for modulating the pathways of interest. This facilitated translational testing in mammalian systems, enabling fast translational application of the results from zebrafish to humans.

### NO-mediated augmentation of liver regeneration

As mentioned above, liver injury induced by acetaminophen exposure is the leading cause of acute liver failure. Identifying factors involved in regulation of embryonic liver growth could reveal conserved pathways with the potential to enhance liver regeneration after injury. We performed a chemical genetic screen in fluorescent reporter embryos (Garnaas et al., 2012), which revealed NO signaling as a novel regulator of liver development (Cox et al., 2014). Despite conservation of the vasoactive effects of NO in the zebrafish (North et al., 2009), it was found not to be the mechanism of action for liver modulation. In contrast, chemical inhibition or knockdown of *S*-nitrosoglutathione reductase (GSNOR), which negatively regulates protein nitrosylation, was shown to mediate the effects of NO on liver growth. To determine conservation of effect in organ regeneration, models of both physical (partial hepatectomy) and chemical (acetaminophen) liver injury were used. Treatment with a novel GSNOR inhibitor (GSNORi) after liver resection in adult fish enhanced cellular proliferation and regeneration. In acetaminophen-exposed larvae and adults, GSNORi significantly prevented hepatocyte necrosis, enhanced proliferation, and improved survival alone and in combination with the current clinical therapeutic intervention, N-acetylcysteine. Significantly, the impact of GSNOR modulation is evolutionarily conserved, as GSNOR knockout mice and GSNORi-treated wild-type mice were similarly protected from acetaminophen-induced liver injury. GSNORi combines hepatoprotective and pro-proliferative properties, and represents a novel therapeutic approach for patients with toxic liver failure.

### PGE2-mediated acceleration of hematopoietic reconstitution

HSC formation and function is highly conserved across vertebrate species; the pathways regulating HSC formation during embryogenesis often maintain a role in HSC maintenance in the adult (North et al., 2004). In particular, RUNX1 function is required for HSC development in all vertebrates examined, including zebrafish, mice and humans (North et al., 2002). To identify novel modulators of HSC formation and homeostasis, we screened a panel of bioactive compounds for effects on runx1+ stem cell induction (North et al., 2007) and identified prostaglandin production as potent HSC regulator. Treatment with a long-acting version of PGE2 (dmPGE2), the most biologically abundant prostanoid (Grosser et al., 2002), consistently increased HSC production, as measured by expression analysis and the use of *in vivo* HSC reporters. In contrast, cyclooxygenase inhibition using both non-selective and specific inhibitors reduced HSC number. Adult irradiation recovery assays showed maintenance of the effect in regenerative repair, and murine embryonic stem cell differentiation studies showed conservation of function across vertebrate species. Long-term hematopoietic repopulation of the murine bone marrow following irradiation injury and limiting-dilution competitive transplantation of PGE2-exposed donor cells revealed increased *in vivo* regenerative potential in mammalian models. Subsequent investigations in zebrafish and mice indicated that PGE2 enhanced HSC function through cAMP-mediated enhancement of Wnt signaling, providing a useful biomarker for translational applications (Goessling et al., 2009). Finally, preclinical studies using human umbilical cord blood cells *in vitro* and in xenotransplantation demonstrated both safety of use with human cells and strong conservation of effect (Goessling et al., 2011).

Following collaborative discussions with transplant physicians and acquisition of toxicity profiles from earlier clinical endeavors, translational application of PGE2 in hematopoietic transplantation therapy clinical trials was approved by the FDA, the first such study to arise from a chemical genetic screening approach in zebrafish. In phase 1 trials primarily designed to establish safety, PGE2-treated HSC samples showed substantial changes in clinical end points, with both predominant engraftment of PGE2-treated cord blood samples and accelerated recovery of the blood counts in patients receiving the transplants compared to historical controls (Cutler et al., 2013); there was no negative impact of PGE2 treatment on donor cell maintenance. A multicenter phase 2 clinical trial further investigating the efficacy of PGE2 treatment for enhancement of HSC function in transplantation therapy is currently ongoing (http://www.clinicaltrials.gov/ct2/show/NCT01627314). In this study, both neutrophil engraftment and bone marrow chimerism will be assessed as primary clinical end points. Together, these examples from our own collaborative investigations indicate that zebrafish can be effectively used as a relevant preclinical therapeutic screening and regenerative model system that enables direct application and efficient translation to the clinical setting.

## Conclusions

Regenerative medicine holds great promise for the alleviation of morbidity and mortality associated with organ failure or injury. Tissue repair and regeneration can be driven by modulation of the pathways that govern stem cell behavior and organ development. The zebrafish has traditionally been an excellent model to study early development and organogenesis, demonstrating high genetic and functional conservation with mammals. Given the inherent connection between developmental pathways and organ repair, this strength combined with a growing list of innovative regenerative models makes the zebrafish an ideal system to study regenerative processes, with the potential to translate relevant findings across species and toward clinical application. In light of the many promising projects mentioned here, developed over such a brief time frame, we anticipate an increasing number of valuable studies and novel therapeutics inspired by and discovered through zebrafish research will be developed in the field of regenerative medicine.
